# Characterization in the rat of the individual tendency to rely on alcohol to cope with distress and the ensuing vulnerability to drink compulsively

**DOI:** 10.1093/braincomms/fcae169

**Published:** 2024-05-14

**Authors:** Lucia Marti-Prats, David Belin

**Affiliations:** Department of Psychology, CLIC (Cambridge Laboratory for Research on Impulsive/Compulsive Disorders), University of Cambridge, Cambridge CB2 3EB, UK; Department of Psychology, CLIC (Cambridge Laboratory for Research on Impulsive/Compulsive Disorders), University of Cambridge, Cambridge CB2 3EB, UK

**Keywords:** alcohol, alcohol use disorder, schedule-induced polydipsia, individual vulnerability, self-medication

## Abstract

Only some vulnerable individuals who recreationally drink alcohol eventually develop the compulsive drinking pattern that characterizes alcohol use disorder. A new frontier in biomedical research lies in understanding the neurobehavioural mechanisms of this individual vulnerability, a necessary step towards developing novel effective therapeutic strategies. Translational research has been hindered by the lack of valid, reliable and robust approaches that enable the study of the influence of the reliance on alcohol to cope with stress or self-medicate negative emotional states on the subsequent transition to alcohol use disorder. We have therefore developed a behavioural task in the rat that enables the investigation of the neural and cellular basis of the exacerbation of the vulnerability to develop compulsive alcohol drinking by the use of alcohol to develop an adjunctive, anxiolytic, polydipsic drinking behaviour in a schedule-induced polydipsia procedure. Hence, in our task, alcohol is introduced in the schedule-induced polydipsia context after several weeks of training with water so that rats are exposed to alcohol for the first time in a distressing context and learn to drink alcohol as a coping strategy. Capitalizing on this protocol, we have consistently been able to identify a subpopulation of rats that were unable to learn to cope with negative states by drinking water and relied on alcohol to do so. This maladaptive reliance on alcohol drinking to cope with distress has been shown to be associated with an exacerbation of the subsequent transition to compulsive drinking. Furthermore, these vulnerable rats reached blood alcohol levels comparable to that of intoxication in humans, thereby developing two key features of alcohol use disorder, namely excessive alcohol intake and compulsive drinking. Altogether, this behavioural task provides a novel and unique tool for the investigation of the neurobehavioural mechanisms underlying the exacerbation of the individual vulnerability to developing compulsive alcohol drinking by the use of alcohol as a strategy to cope with distress, and for the evaluation of the efficacy of potential therapeutic strategies in a personalized medicine approach. This procedure, which focuses on an understudied but key factor of the development of alcohol use disorder, may become widely used as it benefits the fields of alcohol, emotion regulation and stress, the interest in which has substantially increased since the evidence of a profound exacerbation of alcohol use and alcohol-related negative consequences by the distress and social isolation engendered by the various measures implemented worldwide in response to the COVID-19 pandemic.

## Teaser

The reliance on alcohol to cope with stress is associated with an increased susceptibility to develop compulsive drinking in vulnerable individuals. Marti-Prats *et al*. developed a procedure in the rat, based on schedule-induced polydipsia, that enables the identification of these individuals and investigation of the neurobehavioural basis of their vulnerability.

## Graphical Abstract

**Graphical Abstract:**
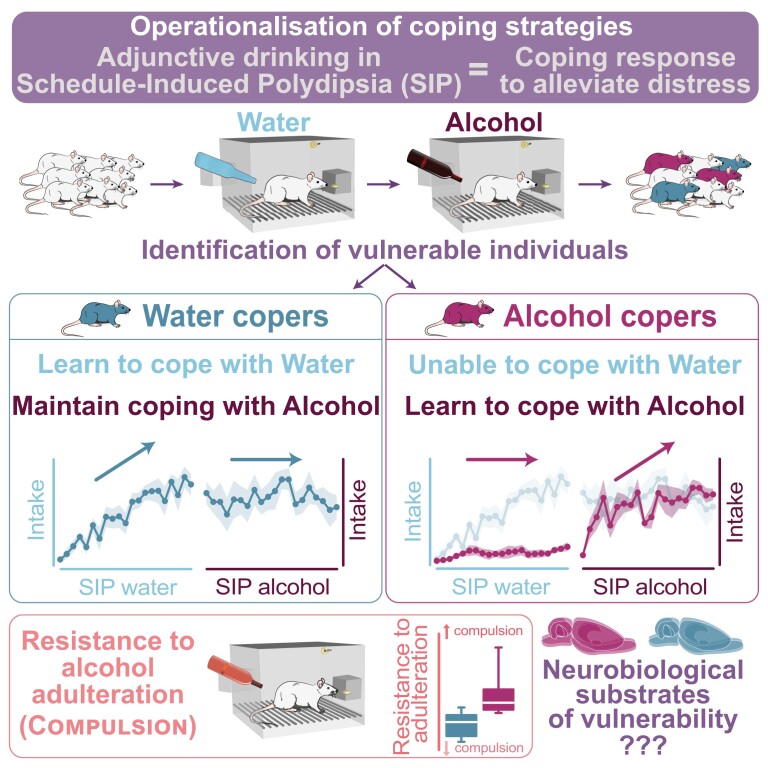

## Introduction to the behavioural task

Behavioural and emotional adaptation to challenging situations is an integral part of emotional homoeostasis necessary for fitness and well-being. Across species, individuals greatly differ in their ability to deploy emotion regulation strategies to cope with distress.^1-8^ Adaptive and maladaptive emotion regulation strategies can be adopted to modulate the magnitude or duration of negative emotional states, resulting in positive or negative outcomes. In general, the implementation of flexible, adaptive strategies results in positive outcomes, such as resilience to stressful events or reduction of negative affect they produce. In contrast, deficits in emotion regulation, such as the implementation of inflexible or maladaptive coping strategies, have been associated with the development and maintenance of psychiatric disorders such as anxiety, obsessive-compulsive (OCD) and substance use disorders (SUD).^3,9-12^

This is consistent with evidence that some individuals who cannot effectively regulate their emotions often turn to alcohol to self-medicate negative states, a maladaptive coping strategy that plays a determining role in their subsequent vulnerability to developing alcohol use disorder (AUD).^13-19^

The increase in problem drinking associated with distress, isolation and dysphoria during the COVID-19 pandemic is the latest evidence that the use of alcohol as a coping mechanism exacerbates the transition to AUD in vulnerable individuals.^20-22^ Given the significant health burden that AUD places on our society,^23^ a better understanding of the psychological and neural basis of the exacerbated vulnerability to develop compulsive alcohol seeking and drinking by the use of alcohol as a self-medication strategy may enable the identification of novel and more effective therapeutic strategies.

Unfortunately, preclinical models of AUD have hitherto failed to operationalize the role played by the individual tendency to rely on alcohol to medicate negative emotional states in the vulnerability to develop compulsive alcohol seeking and drinking. We have therefore designed a novel procedure in rats that enables the identification of the individual tendency to rely on alcohol to cope with negative internal states and the associated increased vulnerability to developing compulsive alcohol drinking, a key feature of AUD.^24-26^ This new procedure capitalizes on a long-established phenomenon, schedule-induced polydipsia (SIP).^27^

Excessive drinking under SIP was initially identified by Falk^27^ when he observed that food—but not water—restricted rats exposed to a fixed time interval schedule of food delivery while having free access to water, drank water to levels that were excessive and far exceeding their homoeostatic needs.^27-29^ Excessive drinking under SIP has since been described in different animal species and humans,^30-37^ under different experimental conditions and using different substitutes to water,^27,32,36,38-42^ including alcohol.^43^ Thus, when water is replaced by alcohol in the SIP procedure, rats drink excessive levels of alcohol, leading to a state of intoxication.^43,44^

This non-physiological excessive scheduled-induced drinking has been classified as an adjunctive behaviour.^28^ Adjunctive behaviours are instrumental coping responses aiming to alleviate distress, here that provoked by the intermittent delivery of food.^28,41,45-49^ These responses decrease the procedure-induced heightened levels of anxiety and stress-related hormones.^41,46,47,49-54^

**Figure 1:**
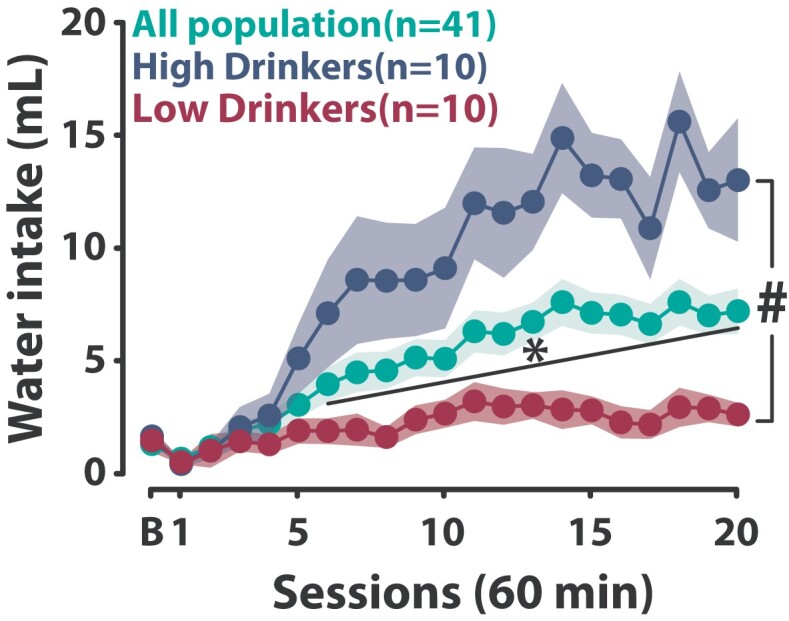
**Individual differences in the development of adjunctive behaviour under schedule-induced polydipsia.** While, at the population level, individuals acquire a coping strategy expressed as an adjunctive polydipsic water drinking response under a fixed time 60-s schedule of food delivery [two-way repeated-measures ANOVA with Greenhouse–Geisser correction, main effect of time: *F*(4.4,176.3) = 21.31, *P* < 0.001, ηp2 = 0.35], some vulnerable individuals (High Drinkers) lose control over their coping strategy over time and develop hyperdipsia, an excessive polydipsic water intake. Other individuals (Low Drinkers) do not acquire a polydipsic coping response with water [two-way repeated-measures ANOVA, main effect of group: *F*(1,18) = 18.67, *P* < 0.001, ηp2 = 0.51; time: *F*(20,360) = 12.22, *P* < 0.001, ηp2 = 0.40 and group × time interaction: *F*(20,360) = 6.97, *P* < 0.001, ηp2 = 0.28]. Data are presented as mean ± SEM. #*P* < 0.001 group × time interaction, *P < 0.001 main effect of time. All data were analysed with a two-way repeated-measures ANOVA with time as within-subject factor and group as between-subject factor, and Greenhouse–Geisser correction when needed. B, baseline session; min, minutes; mL: millilitres.

Like in humans, the majority of individuals who engage in displacement behaviours as a coping strategy tend to maintain relative control over their adjunctive response (Fig. 1), while some vulnerable individuals (High Drinkers, HD) lose control over their polydipsic intake, which becomes excessive and inflexible^44,54-59^ (Fig. 1, see ‘Overview of the procedure’ for a more detailed description). This tendency to develop hyperdipsia, or excessive polydipsic drinking, recapitulates the hallmark features of compulsion, namely the expression of excessive, maladaptive, repetitive behaviours that persist in the face of negative consequences.^24,58,60^ Thus, hyperdipsia has long been suggested to have construct and predictive validity with regard to disorders of compulsion in humans, such as OCD and AUD.^24,58,60-64^

**Figure 2:**
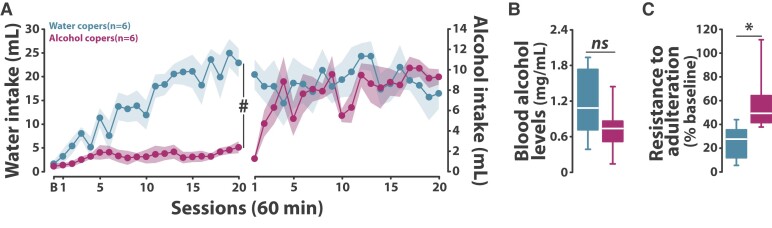
**The reliance on alcohol to develop a coping response facilitates the development of compulsive alcohol drinking (reproduced from Fouyssac *et al*.^44^).** (**A**) While some individuals exposed to a schedule-induced polydipsia procedure progressively developed a polydipsic water drinking response (water copers), others did not [two-way repeated-measures ANOVA, main effect of group: *F*(1,10) = 33.33, *P* < 0.001, ηp2 = 0.77; time: *F*(20,200) = 11.74, *P* < 0.001, ηp2 = 0.54; group × time interaction: *F*(20,200) = 7.96, *P* < 0.001, ηp2 = 0.44] unless they were given access to alcohol (alcohol copers). Thus, the introduction of the opportunity to drink alcohol as a means to cope resulted in the fast development of polydipsic alcohol drinking in these alcohol copers so that alcohol and water copers did not differ in their alcohol drinking behaviour [two-way repeated-measures ANOVA, main effect of group: *F*(1,10) = 1.28, *P* = 0.28; time: *F*(19,190) = 1.23, *P* = 0.23; group × time interaction: *F*(19,190) = 1.44, *P* = 0.11]. (**B**) Water and alcohol coper rats did not differ in their levels of alcohol intoxication [one-way ANOVA, main effect of group: *F*(1,9) = 1.59, *P* = 0.24]. (**C**) However, as compared to the former, the latter persisted in drinking alcohol despite adulteration with quinine [one-way ANOVA, main effect of group: *F*(1,10) = 6.77, *P* = 0.03, ηp2 = 0.40]. Data are presented as mean ± SEM or box plots [medians ± 25% (percentiles) and Min/Max as whiskers]. #*P* < 0.001 group × time interaction, *P < 0.05 alcohol coper different from water coper rats, ns: no significant. All data were analysed with a two-way repeated-measures ANOVA with time as within-subject factor and group as between-subject factor or one-way ANOVA with group as between-subject factor. B, baseline session; min, minutes; mL, millilitres; mg, milligram.

In the context of AUD, beyond its excessive nature, the acquisition of alcohol drinking under SIP may be particularly useful to investigate the neural and cellular basis of drinking alcohol as a self-medication strategy.^62,63,65-67^ Importantly, not all individuals necessarily manage to develop a coping response to the distress generated by intermittent food delivery when water is available (Low Drinkers, Figs 1 and 2). Instead, some of these individuals need alcohol to develop adjunctive drinking (alcohol copers, AC, Fig. 2A). The reliance on alcohol to cope with distress, which is not accompanied with a differential level of alcohol intoxication (Fig. 2B), promotes an exacerbated vulnerability to persist in drinking despite negative consequences, as operationalized by adulteration of alcohol with quinine^44^ (Fig. 2C).

It is this individual tendency to rely on alcohol to cope with stress and the associated increased vulnerability to develop compulsive alcohol drinking that the present procedure has been designed to study. This new procedure, therefore, enables the identification of individuals who are able to cope with stress by drinking water and maintain their established coping strategy when alcohol is introduced (water copers, WC), and individuals who are unable to develop an adjunctive response by drinking water but do so readily with alcohol (AC)^44^ (Fig. 2A).

The present procedure, therefore, provides a novel and unique tool for the investigation of the neurobehavioural mechanisms underlying the exacerbation of the individual vulnerability to develop compulsive alcohol drinking by the use of alcohol as a coping strategy and for the evaluation of the efficacy of potential therapeutic strategies in a personalized medicine approach.

A major advantage of this procedure over those already widely used for the study of AUD lies both in the high level of alcohol intoxication achieved relatively quickly by most animals and in the operationalization of the influence the acquisition of alcohol drinking under negative reinforcement has on the individual vulnerability to develop compulsive alcohol drinking.^68^ Furthermore, the procedure is relatively short and simple enough to be easily associated with other behavioural procedures or in vivo/in vitro measurements/manipulations within longitudinal studies in order to identify the brain mechanisms of the vulnerability to develop AUD. Moreover, the equipment required for the procedure is relatively simple and can be acquired commercially or even 3D printed.

An essential requirement of the procedure is food restriction, which contributes to the distressing properties of intermittent food delivery. This factor could be initially considered a limiting factor as it could be argued that animals are drinking alcohol for its caloric content. However, rats exposed to prolonged SIP alcohol preferred alcohol over dextrose,^69^ while rats receiving similar amount of food as those trained in a SIP procedure and with free access to alcohol showed weight loss but did not increase their alcohol intake.^63,70^ Furthermore, a decrease in alcohol drinking over time would be observed if caloric content overshadows hunger. One potential limitation of the procedure, which is inherent to the study of individual differences, is the requirement of large cohorts of rats to be trained simultaneously in order to obtain samples large enough to reach appropriate statistical power and produce meaningful biological results.

## Objectives

- To investigate the tendency to rely on alcohol to cope with distress.
- To investigate the influence of alcohol drinking as a coping strategy on the subsequent vulnerability to develop compulsive alcohol intake.
- To identify the psychological and neural mechanisms underlying the exacerbation of the individual vulnerability to develop compulsive alcohol drinking by the use of alcohol as a coping strategy.
- To evaluate the efficacy of potential therapeutic strategies in a personalized medicine approach.

## Overview of the procedure

**Figure 3:**
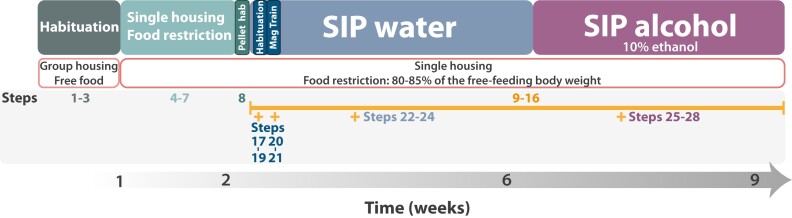
**Timeline of the experimental procedure.** After reception of rats from the supplier or when those bred in-house have reached the required age/body weight, house them in groups with *ad libitum* access to water and chow and allow them to habituate to the vivarium and the experimenter for at least one week (steps 1–3). Following the habituation, restrict the daily amount of food provided until rats reach 80–85% of their theoretical free-feeding body weight, and house them individually. These conditions are to be maintained for the duration of the experiment (steps 4–7). Once the desired body weight has been stable for at least 3 days, give rats some reward food pellets in their home cage the day before starting the training in order to prevent hyponeophagia (step 8). Next day, start the training in the schedule-induced polydipsia (SIP) procedure using either the 60- or 30-min sessions. Start the training with one habituation (steps 9–16 + steps 17–19) and one magazine training session (steps 9–16 + steps 20–21). The total amount of water that each rat drinks over these sessions will provide you with the volume of water ingested to meet their homoeostatic needs while eating 60 reward pellets over the duration of the session. It will be considered as baseline intake. Twenty-four hours after the magazine training session, start the SIP water training stage that consists of at least 20 daily sessions during which rats have free access to a water bottle while 60 reward pellets are delivered under a fixed time (FT) 60- or 30-s schedule over 60- or 30-min sessions, respectively. After each session, calculate the total amount of water consumed by each rat (steps 9–16 + steps 22–24). Twenty-four hours after the last SIP water session, replace the water with 10% ethanol and train the rats in the SIP alcohol procedure for at least 20 sessions. In this stage, rats have free access to the alcohol bottle while 60 reward pellets are delivered under a FT 60- or 30-s schedule over 60- or 30-min sessions, respectively. At the end of each session, calculate the amount of alcohol consumed by each rat (steps 9–16 + steps 25–28). The average of water and/or alcohol consumed over the last two to four sessions will be used to identify different subpopulations of rats (see ‘Overview of the procedure section’ for a detailed description). Pellet hab, pellet habituation; Mag Train, magazine training.

The overview of the procedure is illustrated in Fig. 3.

### Habituation to the vivarium and the experimenter (Habituation)

Upon reception of rats from the supplier, or when those bred in-house have reached the required age/body weight, house them in groups with *ad libitum* access to water and standard chow. Allow rats to habituate to the vivarium and the experimenter for at least one week before starting any procedure (steps 1–3).

### Social isolation and food restriction

Following the habituation, restrict the daily amount of food provided until rats reach 80–85% of their theoretical free-feeding body weight (7–10 days), and house them individually to ensure the welfare of subordinate rats and control their body weight. These conditions are to be maintained for the duration of the experiment (steps 4–7).

### Habituation to the reward pellets (Pellet hab)

Once the desired body weight has been stable for at least 3 days, the day before starting the training, give rats reward food pellets in their home cage in order to prevent hyponeophagia (step 8).

### Training in the SIP procedure

Then, start the training using either the 60-min session fixed time 60-s (FT-60s) schedule of food delivery protocol or the 30-min session FT-30s protocol. Both protocols lead to marked individual differences in the acquisition of alcohol drinking as a coping strategy and high levels of alcohol intake (∼1.5 g/kg) in high alcohol drinker rats (see below):

Habituation session (Habituation): Training starts with one habituation session during which rats have free access to 60 pellets, all available in the food magazine from the beginning of the session, and a water bottle. At the end of this session and the following sessions, the total volume of liquid consumed is calculated as the difference between the weight of the bottle before and immediately after the session. The total volume consumed by each rat during this session is measured to determine the amount of water that each rat drinks to meet its homoeostatic needs while eating sixty 45 mg pellets (steps 9–16 + steps 17–19).

Magazine training (Mag train): The next day, rats are habituated to receiving the 60 reward pellets in the magazine, but this time delivered over the course of the session under a random time schedule, with the water bottle still freely available. The total amount of water drank during this session provides you with the volume of water ingested to meet the homoeostatic needs over the duration of the session (steps 9–16 + steps 20–21). This, alongside the amount of water ingested during the previous session (steps 9–16 + steps 17–19), will be considered as the baseline.

SIP water: SIP training starts the following day with an initial stage of 20 sessions with water. During each session, sixty 45 mg pellets are delivered under a FT-60- or 30-s schedule over 60- or 30-min sessions, respectively (steps 9–16 + steps 22–24).

**Figure 4:**
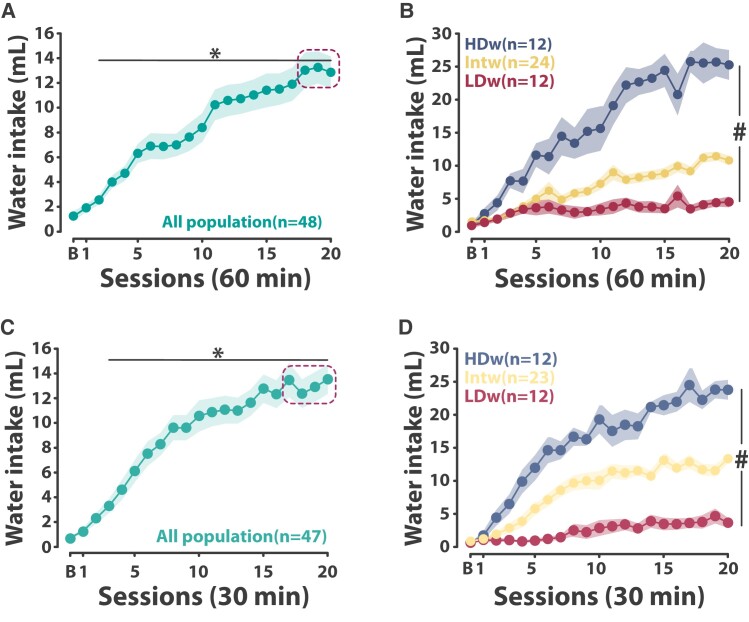
**Identification of individual differences in the development of a coping strategy with water.** (**A**, **C**) At the population level, food-restricted rats exposed to a fixed time 60- or 30-s schedule of food delivery progressively developed adjunctive polydipsic water drinking over 20 sessions [two-way repeated-measures ANOVA with Greenhouse–Geisser correction, main effect of time, 60-min sessions: *F*(3.8,177.8) = 32.82, *P* < 0.001, ηp2 = 0.41; 30-min sessions: *F*(5.3,244.5) = 46.76, *P* < 0.001, ηp2 = 0.50]. Polydipsic drinking became different from baseline drinking from session 3 onwards in both experiments. The average water intake over the last days of training (dashed-line rectangle) was used to subsequently identify differences in the development of a coping polydipsic response with water. (**B**, **D**) Marked individual differences in the propensity to lose control over adjunctive water drinking behaviour as a coping strategy emerged over the 20 sessions of training with High water Drinker (HDw) rats, selected in the upper quartile of the population, developing hyperdipsia, whereas Low water Drinker (LDw) rats, selected in the lower quartile of the population, did not develop a coping strategy with water. The other Intermediate water Drinker (Intw) individuals developed a controlled, stable, adjunctive polydipsic adjunctive drinking [two-way repeated-measures ANOVA with Greenhouse–Geisser correction, 60-min session: main effect of group: *F*(2,45) = 49.16, *P* < 0.001, ηp2 = 0.68; time: *F*(20,900) = 45.51, *P* < 0.001, ηp2 = 0.50; group × time interaction: *F*(15.8,355) = 11.66, *P* < 0.001, ηp2 = 0.34; 30-min session: main effect of group: *F*(2,44) = 70.08, *P* < 0.001, ηp2 = 0.76; time: *F*(20,880) = 58.58, *P* < 0.001, ηp2 = 0.57; group × time interaction: *F*(16,352.03) = 9.43, *P* < 0.001, ηp2 = 0.30]. Data are presented as mean ± SEM. #*P* < 0.001 group × time interaction, *P < 0.05 different from baseline intake. B, baseline session; min, minutes; mL, millilitres. **A** and **B** are reproduced from Fouyssac *et al*.^44^ All data were analysed with a two-way repeated-measures ANOVA with time as within-subject factor and group as between-subject factor and Greenhouse–Geisser correction when needed.

After each session, calculate the total volume of water consumed. Use the average water intake over the last 2–4 days of training (e.g. sessions 18–20), depending on the within/between-subject variability, to select rats in the upper and lower quartiles of the population as High Drinkers (HDw) and Low Drinkers for water (LDw). Rats that do not fall into these categories are deemed Intermediate (Intw) (Fig. 4). HDw rats show a 4–6-fold increase in water intake compared to LDw rats. Thus, HDw rats are vulnerable to develop excessive adjunctive water drinking behaviour whereas LDw rats fail to develop a coping response with water.

SIP alcohol: Twenty-four hours after the last SIP water session, replace water with 10% ethanol and train rats in the SIP alcohol procedure for at least 20 sessions (steps 9–16 + steps 25–28). Calculate the amount of alcohol consumed after each session using the same method as in the previous stage and use the average alcohol intake over the last 2–4 days of training (use the same criteria as in the water stage) to stratify the HDw and LDw populations according to their alcohol intake.

**Figure 5:**
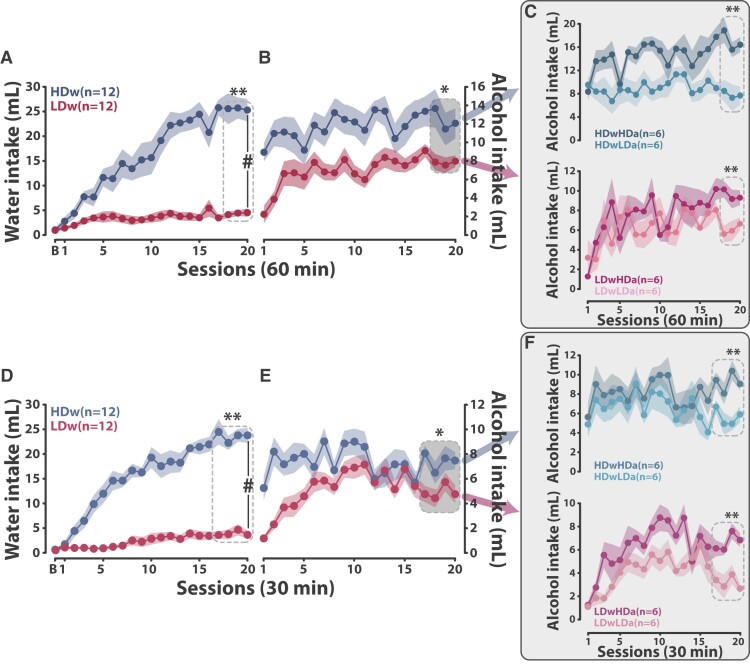
**Identification of individual differences in the tendency to rely on alcohol to develop an adjunctive response.** (**A**, **D**) Rats in the upper and lower quartiles of a population exposed to the schedule-induced polydipsia (SIP) procedure were characterized as High water Drinkers (HDw) and Low water Drinkers (LDw), respectively, based on their water consumption averaged across the last sessions of SIP with water [one-way ANOVA, main effect of group, 60-min sessions: *F*(1,22) = 94.77, *P* < 0.001, ηp2 = 0.81; 30-min sessions: *F*(1,22) = 170.91, *P* < 0.001, ηp2 = 0.89]. HDw rats developed hyperdipsia whereas LDw rats failed to develop a coping polydipsic response with water [two-way repeated-measures ANOVA without or with Greenhouse–Geisser correction, 60-min sessions: main effect of group: *F*(1,22) = 53.97, *P* < 0.001, ηp2 = 0.71; time: *F*(20,440) = 23.96, *P* < 0.001, ηp2 = 0.52; group × time interaction: *F*(20,440) = 16.03, *P* < 0.001, ηp2 = 0.42; 30-min sessions: main effect of group: *F*(1,22) = 133.05, *P* < 0.001, ηp2 = 0.86; time: *F*(20,440) = 34.89, *P* < 0.001, ηp2 = 0.61; group × time interaction: *F*(5.83,128.35) = 18.68, *P* < 0.001, ηp2 = 0.46]. (**B**, **E**) The introduction of alcohol resulted in significant changes in coping behaviour. While HDw rats maintained overall a steady level of polydipsic drinking, now of alcohol, over time some LDw rats acquired a coping response with alcohol and eventually developed polydipsic drinking. While analysis of the average alcohol intake over the last sessions of alcohol training revealed that HDw and LDw rats differed in their consumption of alcohol [one-way ANOVA, main effect of group, 60-min sessions: *F*(1,22) = 6.86, *P* = 0.015, ηp2 = 0.24; 30-min sessions: *F*(1,22) = 7.92, *P* = 0.010, ηp2 = 0.26], (**C**, **F**, bottom panel) a median split applied to each group identified LDw rats that relied on alcohol to acquire a polydipsic coping strategy (Low Drinker water–High Drinker alcohol, LDwHDa) in contrast with others (Low Drinker water–Low Drinker alcohol, LDwLDa) that continued not to show any adjunctive response [one-way ANOVA, main effect of group, 60-min sessions: *F*(1,10) = 21.22, *P* < 0.001, ηp2 = 0.68; 30-min sessions: *F*(1,10) = 22.84, *P* < 0.001, ηp2 = 0.70]. (**C**, **F**, top panel) Similarly, half the HDw rats were revealed to have a level of polydipsic alcohol drinking similar to that they used to show with water (High Drinker water–High Drinker alcohol, HDwHDa), while others drank less alcohol than they did water (High Drinker water–Low Drinker alcohol, HDwLDa) [one-way ANOVA, main effect of group, 60-min sessions: *F*(1,10) = 24.47, *P* < 0.001, ηp2 = 0.71; 30-min sessions: *F*(1,10) = 28.83, *P* < 0.001, ηp2 = 0.74]. HDwLDa and LDwHDa are referred to as water copers (WC) and alcohol copers (AC), respectively. Data are presented as mean ± SEM. #*P* < 0.001 group × time interaction, **P < 0.001 and *P < 0.05 main effect of group. B, baseline session; min, minutes; mL, millilitres. **A** and **B** are reproduced from Fouyssac *et al*.^44^ All data were analysed with a two-way repeated-measures ANOVA with time as within-subject factor and group as between-subject factor and Greenhouse–Geisser correction when needed or one-way ANOVA with group as between-subject factor.

Thus, following a nested design, apply a median split on the alcohol intake of each of the populations to discriminate rats that rely on alcohol to acquire a coping strategy [HD for alcohol (HDa)] and those that do not [LD for alcohol (LDa)]. This leads to the identification of two subpopulations for each initial group: High Drinker water–High Drinker alcohol (HDwHDa) versus High Drinker water–Low Drinker alcohol (HDwLDa, so-called water copers, as in Fig. 2), and Low Drinker water–High Drinker alcohol (LDwHDa, also called alcohol copers, as in Fig. 2) versus Low Drinker water–Low Drinker alcohol (LDwLDa) (Fig. 5). The model relies on the comparison of water coper and alcohol coper rats, which profoundly differ in their tendency to persist in drinking alcohol despite adulteration with quinine even though they have an overall similar exposure to alcohol beforehand, only differing in the motive that underpinned their alcohol drinking. In contrast, HDwHDa drink more alcohol than their peers, so any behavioural or post-mortem comparison with the other groups would be confounded by latent inhibition^71^ and differential exposure to alcohol, while LDwLDa rats show no phenotype of interest in the context of the question at hand.

## Hazards

- Rats: bites, scratches, allergic reactions
- Manual handling: musculoskeletal injuries
- Long experimental procedures: isolation

## Control measures

- A Home Office-approved personal license or similar is required to carry out a procedure on animals in the UK or in other countries
- Your training record must always be up to date for all relevant procedures before initiating a new experiment. If in doubt, consult the NACWO in the UK or the competent authority in other countries
- Make sure you know where the first aid kits are and that you have the first aider’s coordinates
- Wear full personal protective equipment as per standard animal facility regulations
- Ensure that appropriate breaks are taken (to have fresh air and avoid social isolation)
- Ensure that equipment is in good working condition and set up long before you start your experiment

## Materials

**Figure 6:**
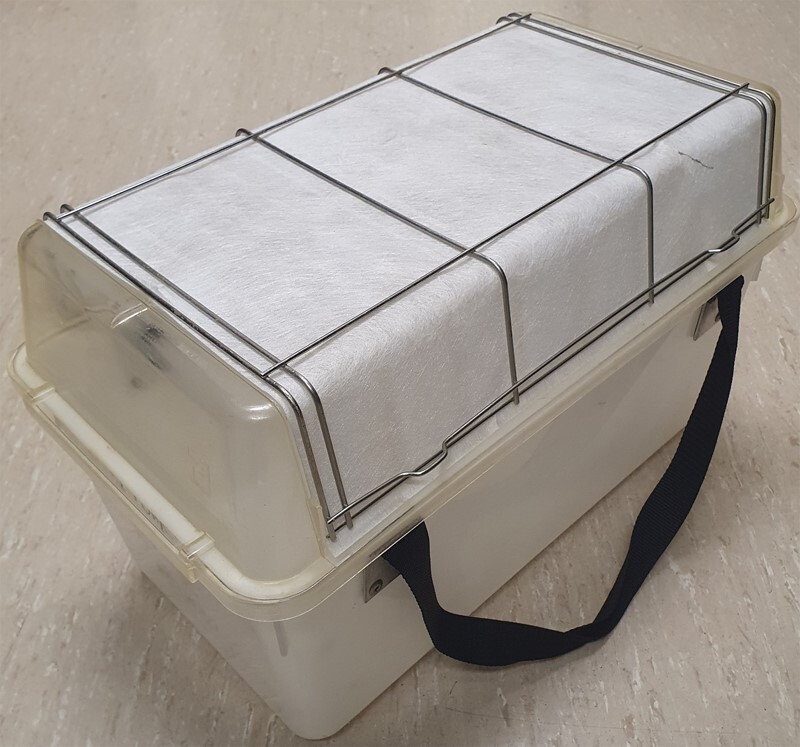
**Carrying box for rats.** Carrying box used in our laboratory.

**Figure 7:**
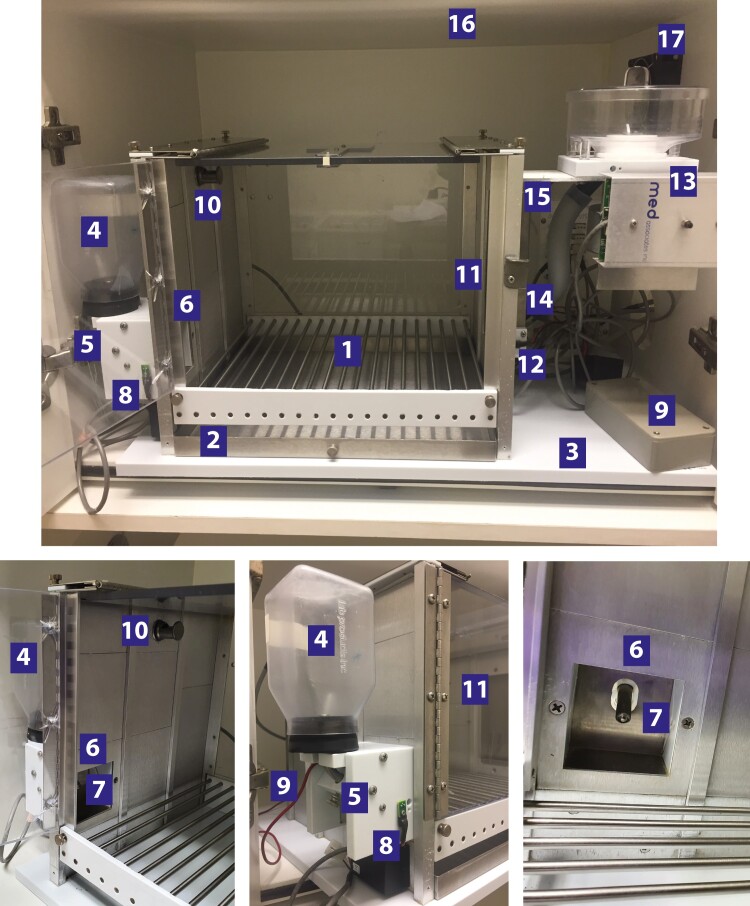
**Schedule-induced polydipsia apparatus.** Description of a standard modular chamber for schedule-induced polydipsia in rats (L × W × H: 32 × 27 × 25 cm) made of stainless steel (side walls) and clear polycarbonate (top, door and rear panels) with a stainless-steel grid floor (1) and stainless-steel waste pan (2), placed on a white polypropylene base (3). The bottle with a curved sipper tube (4) is fitted with the dedicated screw (5) on a magazine that has an open access (6) through which the curved sipper tube (7) protrudes inside the chamber. This bottle magazine, equipped with an infra-red photobeam (8, manufacturer)/contact (9, home-made) lickometer system, is placed at the bottom of the left wall (5 cm above the base). Each chamber is illuminated by a house light (3 W) (10) located at the top of the centre panel of the left wall (22 cm above the base), opposite the food magazine (11) equipped with an infra-red photobeam detection system (5.5 cm above the base) (12). The pellet dispenser (13) is located outside the chamber on the right and delivers the reward pellets to the food magazine (11) through a plastic tube (14). Inputs and outputs are connected to a SmartCtrl panel (15). The apparatus is enclosed within a sound-attenuating cubicle (16) equipped with a fan (17).

- Any outbred rat (usually Sprague Dawley) weighing 250–280 g at the beginning of the experiment
- Standard laboratory chow
- Reward pellets: 45 mg precision pellets (5TUL, TestDiet, USA; Bio-Serv)
- Tap water
- 99.8% ethanol (e.g. Sigma-Aldrich)
- Surface disinfectant for cleaning (e.g. Distel)
- Scale for rats
- Scale for bottles
- Measuring cylinder
- Permanent marker
- Rat-carrying boxes (see Fig. 6)
- SIP apparatus (see Fig. 7):
  ∘ Modular chamber standard for rat (ENV-008CTC, Med Associates, St. Albans, USA)
  ∘ Stainless-steel grid floor (ENV-005, Med Associates, St. Albans, USA)
  ∘ Sound-attenuating cubicle equipped with a fan (ENV-022MD, ENV-025F, Med Associates, St. Albans, USA)
  ∘ Pellet dispenser for rat-45 mg (ENV-203M-45, Med Associates, St. Albans, USA)
  ∘ Plastic tube
  ∘ Pellet receptacle with extra tall opening (ENV-200R2M-6.0, Med Associates, St. Albans, USA)
  ∘ Infra-red photobeam head entry detection system (ENV-254-CB, Med Associates, St. Albans, USA)
  ∘ House light (ENV-215M, Med Associates, St. Albans, USA)
  ∘ Bottle with curved sipper tube + bottle magazine (ENV-251L, Med Associates, St. Albans, USA). For our experiments, we removed the ‘V’ plate provided by the manufacturer.
  ∘ Lickometer system. The product ENV-251L from Med Associates is equipped with a photobeam system. However, we use a custom-made contact lickometer system.
  ∘ Replacement bottles with curved sipper tubes (ENV-250BT, Med Associates, St. Albans, USA)
  ∘ SmartCtrl connection panel (SG-716), interface modules (DIG-716), interface cabinet and appropriate cables (Med Associates, St. Albans, USA)
- Computer equipped with Med-PC software
- Spreadsheet editor software (e.g. Microsoft Excel)
- Data analysis software (e.g. Statsoft STATISTICA, MATLAB and R)
- Data recording sheets

## Procedure

Before starting the procedure, make sure that you have been signed off to perform all the regulated procedures associated with this protocol.

### Habituation to the vivarium and the experimenter (group housing, free food, minimum 7 days)

**Figure 8:**
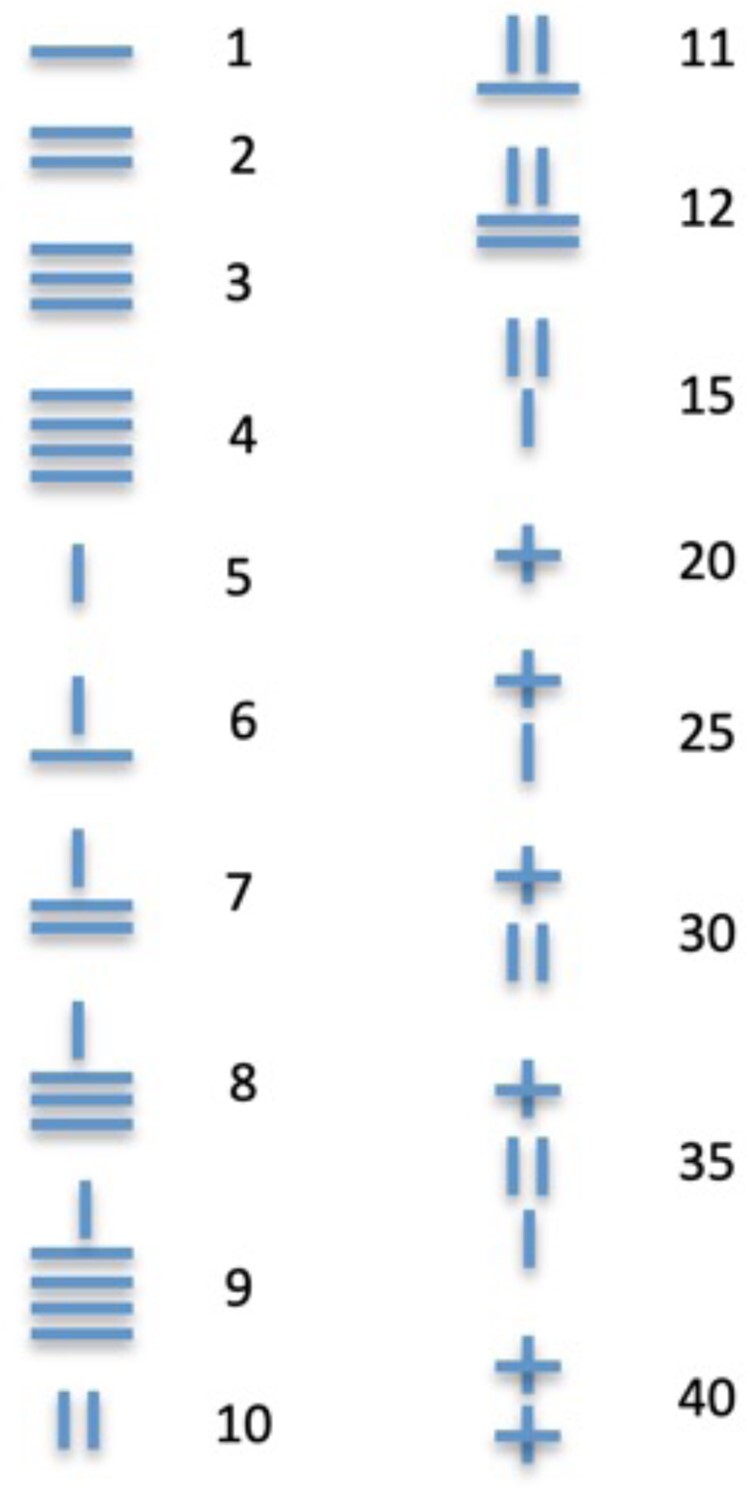
**Rat identification system.** Tail labelling system used in our laboratory to identify rats.

1. Upon reception of 250–280 g rats from the supplier, or when those bred in-house have reached that body weight, house them in groups (2–4 rats per cage) with *ad libitum* access to water and standard chow. The vivarium should be maintained at a constant temperature (22 ± 1°C) and humidity (60 ± 5%) under a 12-h reverse light/dark cycle (lights ON at 19:00) for the entire duration of the experiment.
⇒ The home cage can receive mild enrichment, but this may result in a decrease in the vigour of the polydipsic response. Enrichment of the housing context results necessarily in the relative impoverishment of the training context, which has been demonstrated to exacerbate the vulnerability to develop compulsive cocaine or alcohol intake.^44^
2. Starting at least 2 days after their arrival at the vivarium, habituate the rats to the experimenter by handling each 3–5 min per day, and weigh them at least twice over this period to monitor their growth. Keep the weight record updated within the dedicated spreadsheet (e.g. Excel).
3. Mark the tail of each rat with a unique identification number following the system adopted by your research group (see example in Fig. 8).
⇒ Re-mark the tails throughout the experiment as often as needed to avoid misidentifications.
NB:
⇒ Ensure that the sipper tubes of the home cage bottles are delivering water. Malfunction of the sipper tubes could impinge on feeding and behavioural performance overall.

### Social isolation and food restriction (single housing, rats are food restricted to reach 80–85% of the free-feeding body weight over a period of 7–10 days, then maintained under food restriction until the end of the experimental procedure)

4. At the end of the habituation period, weigh the rats and place them in individual cages with *ad libitum* access to water. Start the food restriction by removing all food from the tray and placing 14 g of standard chow around the time when the feeding will occur on the following days.
⇒ Wherever necessary, follow the animal facility instructions to notify that rats are now under food restriction and socially isolated.
5. From the second day of food restriction until the targeted weight has been reached, weigh the rats daily and tailor the quantity of food each is given to reach and maintain the targeted body weight. For the initiation of weight loss, reducing the daily amount of food to 14–16 g of standard chow should lead to gradual weight loss, although some rats may require additional adjustments in the amount of food provided to reach the 80–85% theoretical free-feeding body weight target.
⇒ Follow the animal facility instructions to record that rats have been fed (i.e. sign the food restriction board and notebook every day).
6. Once the 80–85% of theoretical free-feeding body weight target has been reached, weigh the rats at least 4 days a week and tailor the quantity of food given to each in order to maintain growth.
NB:
⇒ Ensure that the home cage bottles are delivering water and that their sipper tubes are the same for all rats. Whenever possible, use the same sipper tubes as those used in the SIP procedure.
⇒ Weigh the rats at approximately the same time every day with the same scale. When training begins, weigh the rats before the session.
⇒ Keep the Body Weight spreadsheet updated and carefully monitor body weight. Maintaining an optimal level of food restriction and, therefore, body weight is essential for the SIP procedure to induce robust polydipsic drinking.
7. Towards the end of the 7–10 days needed to adjust the body weight of the rats to 80–85% of their free-feeding body weight, check that the Skinner boxes (e.g. light, pellet dispenser and lickometer), bottles and Med-PC software are in perfect working condition; assign each rat to a single Skinner box and assign an identification number to each bottle; practice setting up the bottles in the boxes as loss of liquid during loading and unloading can jeopardize the reliability of the measurement.

### Habituation to the reward pellets (single housing, food restriction, 1 day)

Start this stage, and therefore the training, when the body weight of the rats has been stable at 80–85% of their free-feeding body weight for at least 3 days.

8. The day before the start of training in the SIP procedure, place 20 of the reward pellets (TestDiet 45 mg pellets) used in the SIP procedure in each rat’s home cage in order to avoid hyponeophagia. After 30 min to1 h, feed the rats with standard chow as per the food restriction regime, considering the quantity of food (pellets) already eaten.

### Training in the SIP procedure (single housing, food restriction, at least 42 days)

Follow the general steps for all the training stages and refer to the specific steps for each of the different stages.

Train the rats during the active phase of their circadian cycle.

#### General training steps

9. Weigh the rats and (optional) move their home cages from the housing room to a holding room near the experimental room.
10. Start the software (MED-PC-IV) and ensure that all input/outputs are live and functional.
11. Fill all the bottles with the experimental fluid (i.e. fresh tap water or 10% ethanol, ~250 mL/bottle), weigh the bottles and record the weight as the ‘pre-session value’.
⇒ Ensure that the bottles are well closed and check that the sipper tubes are delivering water before recording the pre-session weight value.
12. Place each bottle in the corresponding Skinner box and secure it with the dedicated screw. Each bottle should be labelled with an identification number that matches the box in which it is installed.
⇒ Ensure that the sipper does not leak and that its ball is well at the tip of the sipper tube. Once the bottle is fixed, dripping should stop after 1–2 drops.
13. Bring the rats to the experimental room in carrying boxes (max 6 rats/box), place each rat in its assigned box and start the adequate training session by uploading either a pre-recorded macro or the corresponding program (see next section, ‘Specific steps for each stage of training’). The start of the session is signalled by turning ON of the house light and the cubicle fan; head entries in the food magazine and licks are recorded throughout the session. At the end of the 60/30-min session, the house light and fan are switched OFF.
⇒ Ensure that each rat is in the correct Skinner box and both boxes and cubicles are properly closed.
⇒ Ensure that the pellet dispensers are functioning properly before starting a new session.
⇒ Ensure that all Skinner boxes are functioning properly. At the beginning of the session, the program should start acquiring data for all boxes (magazine entries are usually the first event to be recorded). If the program is not recording data for some of the boxes, check through the viewing port of the external cubicle that the house light is ON, which is evidence that the session has started but the rat has not performed a task-relevant action yet; otherwise, check that the cables are properly connected.
14. Immediately after the termination of the session, remove the rats from the boxes and bring them back to their home cages with carrying boxes. Then, remove the bottles, weigh them and record the weight as the ‘post-session value’. Calculate the liquid intake for each rat as the difference between the post- and pre-session weights.
Record the total licks and magazine entries displayed on the computer screen for each rat (in case anything goes wrong with the recording of the data or the hard drive of your machine).
15. At least 1 h after each session, feed the rats with the appropriate quantity of food as per the food restriction regime.
⇒ Follow the animal facility instructions to record that rats have been fed (i.e. sign the food restriction board and notebook every day).
16. After the last session, move the rats back to their housing room (optional, only if step 9 is carried out). Daily, empty the bottles and clean them, wipe down the Skinner boxes, waste pans and carrying boxes. Turn off all electrical equipment (computer, Med-PC interface).
⇒ When cleaning the Skinner boxes, an excessive amount of liquid under the waste pan may indicate bottle spillage or rat playing with the spout. Make a note of it in your log book and address the issue (see ‘Troubleshooting’ section).
NB:
⇒ It is recommended to record a macro for each stage of the experimental procedure before starting the training.
⇒ Never change the assignment of the Skinner box, as slight changes in the environment can have unexpected behavioural consequences.
⇒ The time at which the rats are trained and fed should be kept constant throughout the experiment.
⇒ Ensure that the reward pellets are delivered properly. The dispenser may be blocked due to the moisture inside the box or cluttering. If the pellets are undelivered, rats will not be exposed to the anxiogenic properties of the task.
⇒ Update the rat’s weight and session information (liquid intake, licks and magazine entries) daily in a dedicated Excel spreadsheet. This will help with the curation of the data prior to making them available for open access in the future!
⇒ Do not mark the tail immediately before the training sessions, as rats urinate profusely during the training sessions, which would cause loss of fresh labelling.
⇒ If required, follow the facility instructions to notify that rats have started the behavioural procedure.

#### Specific steps for each stage of training

##### Habituation: water (1 day, 24 h after the habituation to the reward pellets)

17. After placing the water bottles in the boxes (step 12) and before initiating the session, place 60 reward pellets manually in the food magazine of each Skinner box or upload a Med-PC program to deliver 60 pellets in the food magazine (Supplementary file 1: SIP Free delivery 60 pellets).
18. Start the session (step 13) by uploading the appropriate Med-PC program for each rat (Supplementary file 2: SIP baseline/Supplementary file 3: SIP baseline 30 min, 60- or 30-min session, respectively). The start of the session is signalled by the turning ON of the house light and the cubicle fan, both remaining ON throughout the session (60- or 30-min session). No pellets are delivered in this session.
19. The volume of water consumed by each rat over this session (step 14) determines the amount of water each individual drinks to meet homoeostatic needs associated with the ingestion of sixty 45 mg pellets.

##### Magazine training: water (1 day, 24 h after the habituation session)

20. Start the session (step 13) by uploading the appropriate Med-PC program for each rat (Supplementary file 4: Magazine training VI 60 SIP/Supplementary file 5: Magazine training VI 30s 60p 30 min, 60- or 30-min session, respectively). The start of the session is signalled by the turning ON of the house light and the cubicle fan, both remaining ON throughout the session (60- or 30-min session); 60 reward pellets are delivered under a random time schedule of reinforcement.
21. The volume of water consumed (step 14) provides information about the volume of water each individual drinks to meet homoeostatic needs related to the ingestion of 45 mg pellets over the specific duration of the session.

##### SIP water (at least 20 days, session 1 being carried out 24 h after the magazine training session)

22. Use the appropriate Med-PC program (step 13) to start the training session (Supplementary file 6: SIP/Supplementary file 7: SIP FI 30 min 60p, 60- or 30-min session, respectively). The start of the session is signalled by the turning ON of the house light and the cubicle fan followed by the delivery of a pellet, subsequently up to 60 pellets will be delivered following a FT-60- or 30-s schedule (60- or 30-min session); both house light and fan remain ON for the entire session (60- or 30-min session).
23. Calculate the total amount of water consumed for each rat daily (step 14).
24. Train the rats in the SIP procedure with water for at least 20 sessions.

##### SIP alcohol (at least 20 days, session 1 is 24 h after the last water session)

25. Prepare the 10% ethanol solution needed to fill all the bottles by dilution of pure ethanol with tap water, either the day prior to the session or before weighing the rats on the day of the session (step 9).
26. Start the session (step 13) by uploading the same Med-PC program as in the training with water (60- or 30-min session) (step 22).
27. Calculate the alcohol consumption for each rat daily (step 14).
28. Train the rats in the SIP procedure with alcohol for at least 20 sessions.

## Data

### Data collection

Record the following variables for each animal and each session in the recording sheets and subsequently transfer the data to the software of election (e.g. Excel or R):

- Total number of entries in the food magazine: provided by the Med-PC software.
- Total amount of liquid consumed: the difference between the weights of the bottle before and after the session. Collected manually by weighing the bottles.
- Total number of licks: provided by the Med-PC software.

While the characterization of the individual tendency to rely on alcohol to cope with stress and the ensuing increased vulnerability to develop compulsion does not rely on the microdissection of licking behaviour (e.g. bouts and bouts per inter-pellet interval), such analysis can prove useful in future investigations, especially with regard to the investigation of the habitual nature of the behaviour.^59,72,73^ Thus, relevant variables for analysis of the licking pattern can be extracted from the Med-PC raw data files using a bespoke script.

### Characterization of the population

#### SIP water

At the end of the SIP water training, calculate the average water consumption over the last 2–4 days of training, depending on the within/between-subject variability, and use these data to stratify the population and identify individuals as either (Figs 4 and 5):

- High water Drinkers (HDw, rats that develop an excessive adjunctive drinking response with water) = upper quartile of the population; or
- Low water Drinkers (LDw, rats that do not acquire a coping response with water) = lower quartile of the population.

#### SIP alcohol

At the end of the SIP alcohol training, calculate the average alcohol consumption over the last 2–4 days of training (use the same criteria as in the water training) for the HDw and LDw subpopulations and apply a median split on each of the populations to discriminate rats that rely on alcohol to acquire a coping strategy with alcohol [HD for alcohol (HDa)] and those that do not [LD for alcohol (LDa)] (Figs 4 and 5):

- High water Drinkers–High alcohol Drinkers (HDwHDa) = upper quartile for water intake + above the median for alcohol intake in the HDw subpopulation;
- High water Drinkers–Low alcohol Drinkers (HDwLDa, referred to as ‘water copers’, see Fig. 2) = upper quartile for water intake + below the median for alcohol intake in the HDw subpopulation;
- Low water Drinkers–High alcohol Drinkers (LDwHDa, referred to as ‘alcohol copers’, see Fig. 2) = lower quartile for water intake + above the median for alcohol intake in the LDw subpopulation; and
- Low water Drinkers–Low alcohol Drinkers (LDwLDa) = lower quartile for water intake + below median for alcohol intake in the LDw subpopulation.

## Additional steps

### Resistance to adulteration

In order to further test the compulsive nature of polydipsic alcohol drinking, at the end of the SIP alcohol training, the individual tendency to persist in drinking alcohol despite adulteration with quinine can be assessed.^74-78^ The day of the test, dissolve quinine hydrochloride dihydrate (Sigma-Aldrich) in the 10% ethanol solution at a final concentration of 0.1 g/L,^44,74,79^ fill the bottles with the adulterated ethanol solution and proceed as usual (steps 9–16 + 26–27).

The individual propensity to persist in drinking alcohol despite adulteration can be calculated as the ratio between ‘alcohol + quinine’ intake over baseline alcohol intake (average intake over the last two to four sessions of SIP alcohol prior to the test).

### Blood sampling

At different stages of training or at the end of the procedure, blood samples can be collected to measure blood alcohol levels or other parameters of interest, such as stress hormones. The most appropriate blood extraction technique will be selected based on the experimental procedure timeline and/or parameter to be assessed.

If blood samples are going to be collected after the SIP session, staggering the start of the session between rats is recommended. The delay between sessions will depend on the blood sampling technique and the skills of the researcher. Before performing the procedure, habituate the rats to the sequential initiation of the sessions, as even slight changes in the procedure can cause unintended behavioural consequences.

Refer to the appropriate standard operating procedure (SOP) at your institution for further details. Competency should be demonstrated before undertaking these procedures.

### Pharmacological strategies or viral-based manipulations

In order to identify the neural mechanisms underlying the individual tendency to use alcohol as a coping strategy and the ensuing exacerbation of the development of compulsivity and/or to evaluate the efficacy of potential therapeutic strategies, pharmacological or viral-based manipulations can be used during the first steps of the SIP training, or once the adjunctive drinking response has been established. When systemic or intra-cerebral manipulations are needed, habituate the rats to the manipulation (e.g. injection using vehicle solution and cable connection) for several days prior to the test day/days. The habituation period will depend on the impact such manipulation has on behaviour but try to do as few habituation sessions as possible.

When surgical procedures are needed in the middle of the training, after the recovery period, allow the rats to recover their pre-surgery intake levels before continuing with the experimental schedule.

Refer to the appropriate SOP at your institution for further details. Competency should be demonstrated before undertaking these procedures.

## Troubleshooting

### Excessive weight loss

It can be due to problems with the water bottle in the home cage not delivering water. Ensure that rats have actual access to water in their home cage. Furthermore, make sure rats are being weighed at approximately the same time every day, with the same scale, and that the daily amount of food they have received has been tailored to their actual body weight. If the water bottle has been verified not to have a problem and the rats have been weighed properly, contact the vet.

### Rat stops drinking or unstable drinking behaviour during training

These can be attributable to:

- Malfunction of the pellet dispenser: Clean the pellet dispenser and dispenser tube regularly to prevent any cluttering of the system. Additionally, check that pellets are delivered prior to starting each session.
- Malfunction of the sipper tube: Check the sipper tubes are delivering water after filling the bottles and before recording the pre-session weight value. Furthermore, ensure that the ball is at the tip of the sipper tube when the bottle is placed in the Skinner box. If faulty, replace the sipper tube.
- Unstable or inadequate weight: Weigh the rats at approximately the same time every day, monitor their body weight daily, and adjust the food according to their body weight.
- Fight between rats in the carrying box before starting the session: Do not place the rat inside a carrying box when moving the rats from the holding room to the experimental room.
- House light failure: Check the house light of all the Skinner boxes every day and replace it whenever needed.

### Unusually excessive liquid consumption

Unusually excessive fluid consumption compared to the previous days could be due to a spillage of the experimental liquid. If that is the case, an excessive amount of liquid may be found under the waste pan.

The possible reason for the spillage may be a leaking bottle. Check the bottle to confirm that it is tightly closed and check the rubber and sipper tube to confirm that there are no leaks. If leaking occurs, replace the rubber and/or sipper tube. Alternatively, and on some occasions, rats may play with the spout, leading to excessive spillage of liquid that is not consumed. If this behaviour is confirmed, the animal should be excluded, at least for the sessions during which the behaviour occurs.

### Software fails to start acquiring data for some boxes

This may be due to an electrical problem. Check that all the wires and cables are properly connected.

### Lickometer malfunction

This can be attributable to:

- Electrical problem: Check that all the wires and cables are properly connected.
- Salt deposits: Clean all the pieces of the bottle magazine periodically to avoid salt deposits.
- Water/alcohol spillage: Dry the lickometer environment periodically to avoid system malfunction.
- Damaged sipper tube: Prolonged use of the sipper tube leads to its erosion and a faulty contact between the screw that secures the bottle and the sipper tube, which may cause lickometer malfunction. Move the bottle and fix it on an undamaged part of the sipper tube or replace the sipper tube.

### Barbering

Some rats may develop excessive grooming due to the distress triggered by the procedure. Monitor the rat, and if signs of skin irritation of inflammation develop, contact the veterinarian.

## Supplementary Material

fcae169_Supplementary_Data

## Data Availability

Data sharing is not applicable to this article as no new data were created or analysed.
